# Rapid Genomic Characterization and Global Surveillance of *Klebsiella* Using Pathogenwatch

**DOI:** 10.1093/cid/ciab784

**Published:** 2021-12-01

**Authors:** Silvia Argimón, Sophia David, Anthony Underwood, Monica Abrudan, Nicole E Wheeler, Mihir Kekre, Khalil Abudahab, Corin A Yeats, Richard Goater, Ben Taylor, Harry Harste, Dawn Muddyman, Edward J Feil, Sylvain Brisse, Kathryn Holt, Pilar Donado-Godoy, K L Ravikumar, Iruka N Okeke, Celia Carlos, David M Aanensen, NIHR Global Health Research Unit on Genomic Surveillance of Antimicrobial Resistance, Johan Fabian Bernal, Alejandra Arevalo, Maria Fernanda Valencia, Erik C D Osma Castro, Geetha Nagaraj, Varun Shamanna, Vandana Govindan, Akshata Prabhu, D Sravani, M R Shincy, Steffimole Rose, K N Ravishankar, Anderson O Oaikhena, Ayorinde O Afolayan, Jolaade J Ajiboye, Erkison Ewomazino Odih, Marietta L Lagrada, Polle Krystle V Macaranas, Agnettah M Olorosa, June M Gayeta, Melissa Ana L Masim, Elmer M Herrera, Ali Molloy, John Stelling

**Affiliations:** 1 Centre for Genomic Pathogen Surveillance, Wellcome Genome Campus, Hinxton, Cambridge, United Kingdom; 2 Centre for Genomic Pathogen Surveillance, Li Ka Shing Centre for Health Information and Discovery, University of Oxford, Old Road Campus, Oxford, United Kingdom; 3 Milner Centre for Evolution, University of Bath, Bath, United Kingdom; 4 Institut Pasteur, Biodiversity and Epidemiology of Bacterial Pathogens, Paris, France; 5 Department of Infectious Diseases, Central Clinical School, Monash University, Melbourne, Victoria, Australia; 6 London School of Hygiene and Tropical Medicine, London, United Kingdom; 7 Colombian Integrated Program for Antimicrobial Resistance Surveillance (Coipars), CI Tibaitatá, Corporación Colombiana de Investigación Agropecuaria (AGROSAVIA), Tibaitatá–Mosquera, Cundinamarca, Colombia; 8 Central Research Laboratory, Kempegowda Institute of Medical Sciences, Bengaluru, India; 9 Department of Pharmaceutical Microbiology, Faculty of Pharmacy, University of Ibadan, Oyo State, Nigeria; 10 Antimicrobial Resistance Surveillance Reference Laboratory, Research Institute for Tropical Medicine, Muntinlupa, The Philippines

**Keywords:** antimicrobial resistance, epidemiology, genomic surveillance, *Klebsiella*, Pathogenwatch

## Abstract

**Background:**

*Klebsiella* species, including the notable pathogen *K. pneumoniae*, are increasingly associated with antimicrobial resistance (AMR). Genome-based surveillance can inform interventions aimed at controlling AMR. However, its widespread implementation requires tools to streamline bioinformatic analyses and public health reporting.

**Methods:**

We developed the web application Pathogenwatch, which implements analytics tailored to *Klebsiella* species for integration and visualization of genomic and epidemiological data. We populated Pathogenwatch with 16 537 public *Klebsiella* genomes to enable contextualization of user genomes. We demonstrated its features with 1636 genomes from 4 low- and middle-income countries (LMICs) participating in the NIHR Global Health Research Unit (GHRU) on AMR.

**Results:**

Using Pathogenwatch, we found that GHRU genomes were dominated by a small number of epidemic drug-resistant clones of *K. pneumoniae*. However, differences in their distribution were observed (eg, ST258/512 dominated in Colombia, ST231 in India, ST307 in Nigeria, ST147 in the Philippines). Phylogenetic analyses including public genomes for contextualization enabled retrospective monitoring of their spread. In particular, we identified hospital outbreaks, detected introductions from abroad, and uncovered clonal expansions associated with resistance and virulence genes. Assessment of loci encoding O-antigens and capsule in *K. pneumoniae*, which represent possible vaccine candidates, showed that 3 O-types (O1–O3) represented 88.9% of all genomes, whereas capsule types were much more diverse.

**Conclusions:**

Pathogenwatch provides a free, accessible platform for real-time analysis of *Klebsiella* genomes to aid surveillance at local, national, and global levels. We have improved representation of genomes from GHRU participant countries, further facilitating ongoing surveillance.

The *Klebsiella* genus, which belongs to the *Enterobacteriaceae* family, comprises several species that cause opportunistic infections in hospital and community settings [1, 2]. *Klebsiella* species are found in the environment, and commonly contaminate healthcare environments and medical equipment [3, 4]. They also frequently colonize the intestinal tract and other mucosal surfaces of humans, which can serve as reservoirs for infection [1, 5, 6]. Infections occur most commonly in the elderly, neonates, and in immunocompromised individuals, and include urinary tract infections, pneumonia, bloodstream infections, and sepsis [7, 8]. By far the most clinically significant member of the genus is *Klebsiella pneumoniae* [9]. However, other species including *K. quasipneumoniae*, *K. variicola*, and *K. oxytoca* are also notable pathogens [10–12].

In recent years, the prevalence of infections caused by *K. pneumoniae* that are multidrug resistant has risen sharply [13]. Increasing resistance levels have largely been driven by the emergence of strains producing extended-spectrum β-lactamase (ESBL) and carbapenemase enzymes, which are typically plasmid-encoded. ESBLs confer resistance to third-generation cephalosporins and monobactams, while carbapenemases result in resistance to almost all β-lactams including carbapenems [14, 15]. In the community, hypervirulent *K. pneumoniae* can cause severe infections due to the production of specific virulence factors including siderophores and the capsule expression regulator RmpA [16]. Moreover, infections and outbreaks involving strains with both multidrug resistance and hypervirulence have now also been reported, leading to concern over a potential rise of serious untreatable infections [17, 18].

Increasing constraints around treatment options for multidrug-resistant *Klebsiella* infections, and accompanying rise in hypervirulence, have led to an urgent need for novel drugs. Some have reached the market recently, including ceftazidime-avibactam and plazomicin, but more are needed. There is also renewed interest in the development of a preventative vaccine for *K. pneumoniae* infections [19, 20]. Potential vaccine candidates include K-antigens belonging to the bacterial capsule polysaccharide (CPS) and O-antigens comprising the outermost part of the lipopolysaccharide (LPS) [21]. However, these antigens are variable and may differ across geographic regions or infection types, underlining the need for seroepidemiology.

Pathogen surveillance provides a powerful tool for understanding the evolution and spread of resistant bacteria and defining their clinically relevant features [22]. With more widespread adoption of genomic approaches, there is a growing need for tools that streamline whole-genome sequencing (WGS) analyses, circumvent the need for user expertise in bioinformatics, and deliver results for public health utility. Here we describe the *Klebsiella* scheme of the web application Pathogenwatch, which incorporates community-driven tools together with additional functionality to provide detailed typing and phylogenetic analyses of *Klebsiella* isolates, and integration of genomic and epidemiological data [23, 24]. We illustrate features of Pathogenwatch by analyzing *Klebsiella* genomes from 4 low- and middle-income countries (LMICs) participating in the National Institute for Health Research Global Health Research Unit (GHRU) on Genomic Surveillance of Antimicrobial Resistance (AMR). We also demonstrate how it can aid decision making in real time at local and global scales and inform the choice and effectiveness of key interventions such as vaccines.

## METHODS

### Assembly and Curation of Public *Klebsiella* Genomes for Pathogenwatch

We identified 18 319 samples in the European Nucleotide Archive (ENA) labeled either “*Klebsiella*” or “*Raoultella*” (a closely related genus that is not phylogenetically separate from *Klebsiella*; henceforth, included within *“Klebsiella”*) with paired-end Illumina sequence data and geolocation data, as of 3 August 2020. De novo assembly using the raw sequence data was attempted using a SPAdes pipeline, resulting in the assembly of 97.1% (17 783/18 319) of the samples [25]. Various quality-control (QC) metrics were used to discard assemblies of poor quality (Supplementary Tables 1 and 2).

Ninety-three percent (16 537/17 783) of assemblies passed these QC criteria and were imported into Pathogenwatch as public genomes. Metadata for these samples (Supplementary Table 3) were downloaded via the ENA application programming interface (API), curated, and linked to the assemblies in Pathogenwatch.

### Pathogenwatch Features Tailored to *Klebsiella* Species

#### Species Determination

The Speciator tool assigns species by comparing assemblies to genomes within a reference library via Mash [26, 27]. This library comprises a manually curated set of reference assemblies tailored for *Klebsiella* and other *Enterobacteriaceae* species from Kleborate (version 2.0.1 at the time of writing) [28].

#### Genomic Characterization

Multi-locus sequence typing (MLST) and core genome MLST (cgMLST) are performed using the allelic and profile definitions from databases hosted via the BIGSdb platform at Institut Pasteur [29]. Resistance and virulence loci, K- and O-loci, and *wzi* genes are typed in Pathogenwatch via an implementation of Kleborate (version 2.0.1 at the time of writing). K- and O- antigen biosynthesis locus typing is achieved using Kaptive [30, 31], while the *wzi* genes are defined using the BIGSdb-Pasteur platform [32]. Plasmid replicons are identified using Inctyper (currently version 0.0.4), with the *Enterobacteriaceae* database (11 May 2020 version) from PlasmidFinder [33, 34].

#### Phylogenetic Analyses

The pan-genome tool Roary was used previously to identify 2539 genes present in 95% or more of genomes from each species within a European collection of *K. pneumoniae* species complex isolates (*K. pneumoniae*, *K. quasipneumoniae*, *K. variicola*, *K. quasivariicola*) [22, 35]. To further define a set of core genes in *K. pneumoniae* for phylogenetic analyses within Pathogenwatch, we first merged any matches to the 2539 genes that were overlapping in any of 13 diverse *K. pneumoniae* reference genomes to form pseudo-sequences (Supplementary Table 4). We then removed any genes/pseudo-sequences that were paralogous (ie, matched another with >80% nucleotide identity and an e-value of <1e-35 via BLASTn) and any that were absent or incomplete in 1 or more references [36]. The core gene library used in Pathogenwatch thus comprised the remaining 1972 genes (or pseudo-sequences). These are queried to generate pairwise single nucleotide polymorphism (SNP) distances between genomes, which are used to construct neighbor-joining trees.

### Whole-Genome Sequencing and Assembly of GHRU Isolates

Laboratories in 4 participant countries of the GHRU obtained 1706 *Klebsiella* genomes from isolates collected in 2013–2019 (Colombia, n=589; India, n=347; Nigeria, n=164; Philippines, n=606) [37–40]. Of the samples from the Philippines, 342 of 606 (56.4%) have been previously reported [41]. Raw sequence data were assembled and quality-checked as described in this Methods section. Ninety-six percent (1636/1706) of assemblies passed QC criteria and were analyzed with Pathogenwatch (Supplementary Tables 5 and 6). Of these 1636 isolates, 1625 were from human clinical samples and 11 from environmental samples (Nigeria only). Raw data have been deposited in the ENA under study accessions ERP112087, ERP112088, ERP112089, ERP112091, and ERP019480.

## RESULTS AND DISCUSSION

### User Upload and Characterization of *Klebsiella* Genomes Using Pathogenwatch

**Figure 1.**
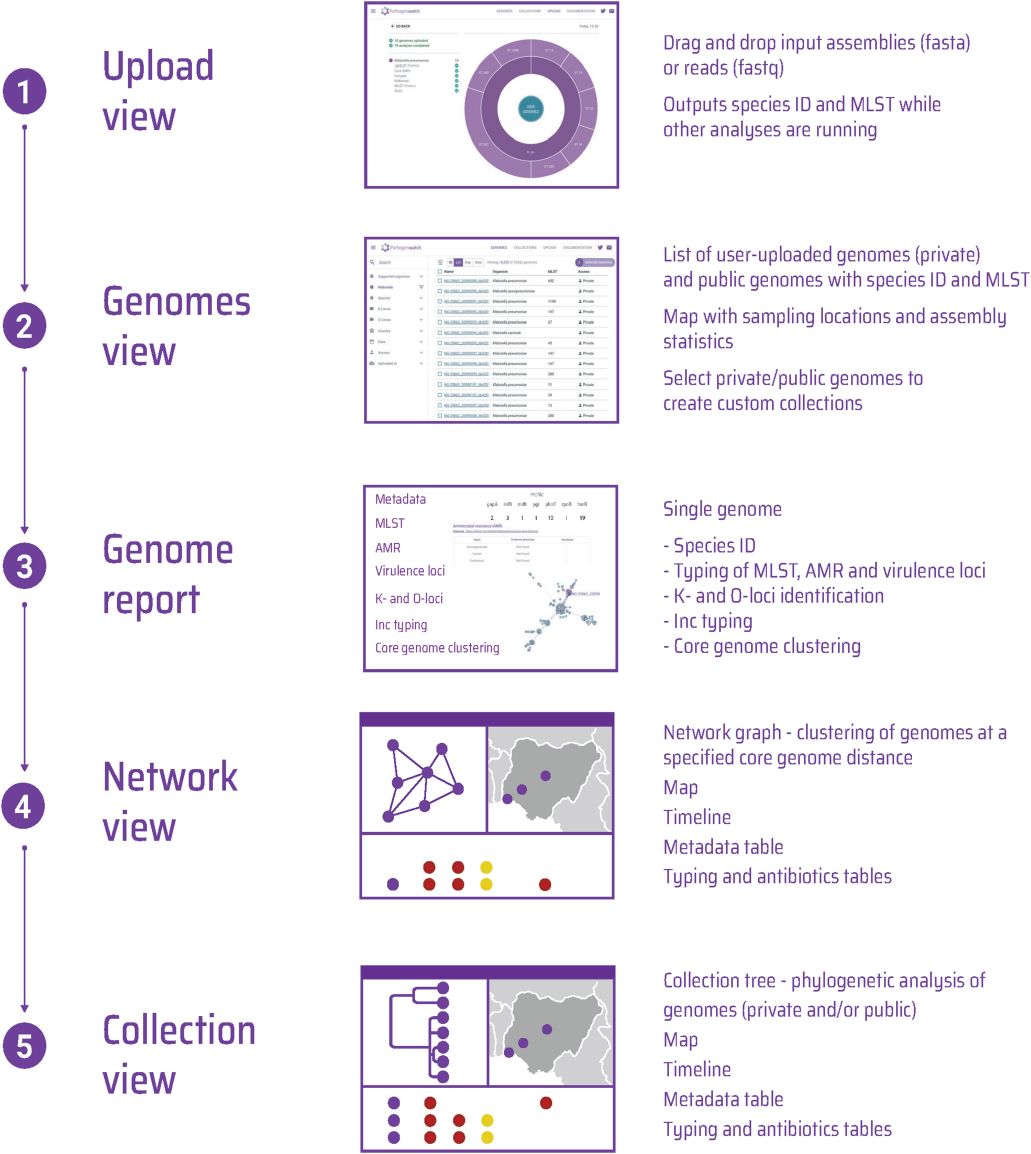
Overview of the analytical processes performed on *Klebsiella* genomes and the available visualizations in Pathogenwatch. Abbreviations: AMR, antimicrobial resistance; MLST, multi-locus sequence typing.

Here we present features of the Pathogenwatch application (https://pathogen.watch/) that are tailored to genomic analysis of *Klebsiella* (including the closely related *Raoultella* genus). Users can upload either assemblies or raw sequence reads to Pathogenwatch, the latter of which will be assembled via a SPAdes pipeline [25]. Assemblies identified by Pathogenwatch as belonging to *Klebsiella* are then subjected to specific analytic pipelines (Figure 1 ). These include MLST and cgMLST for species with available schemes, identification of resistance genes, virulence loci, capsule and O-antigen biosynthesis loci, and replicon typing (Supplementary Table 7).

As standard in Pathogenwatch, users can browse public and/or uploaded genomes. Public genomes include 16 537 high-quality *Klebsiella* genomes with geolocation data (Supplementary Table 3, Supplementary Figure 1). All metadata and results from the analytic pipelines can be viewed and downloaded for an individual genome in a “Genome report” or collectively for multiple selected genomes from the “Genomes” page. Within the Genome report, a clustering tool can be used to rapidly identify the most closely related genomes (from all public and uploaded genomes) to a genome of interest based on cgMLST allelic differences. Users can generate an interactive network visualization of genomes clustered within a particular allelic threshold.

We have also developed the ability for users to generate a phylogenetic tree comprising multiple selected genomes of *K. pneumoniae* (comprising user and/or public genomes). The tree is functionally integrated in the “Collection” view with a map and timeline, showing the locations and sampling dates of genomes if provided, and results from all analytic pipelines. This visualization enables the user to interactively explore the data, while the tree and all other data from individual collections can be downloaded in standard formats.

All sequence data and metadata uploaded by users remain private to their accounts. Genomes grouped into collections are also kept private by default, although they can be shared with collaborators via a URL. There is also an option for users to integrate confidential metadata into visualizations locally within the browser, without uploading data to the Pathogenwatch server.

Pathogenwatch, as well as the integrated tools, is being actively developed and will be updated periodically to provide the latest typing information (including new resistance and virulence mechanisms), newly available public genomes, and other features. The modular architecture of Pathogenwatch also enables integration of new analytics. Detailed descriptions of all of the above processes can be found in the documentation [42].

Below we describe the utility of Pathogenwatch for epidemiological surveillance with 1636 isolates collected from 4 laboratories linked to national or countrywide networks in Colombia, India, Nigeria, and the Philippines (Supplementary Table 6) [43]. All 4 countries have been previously underrepresented in genomic surveillance efforts (Table 1) despite previous estimates of significant burden of *Klebsiella* infections in those regions [44].

**Table 1.** Distribution of *Klebsiella pneumoniae* Genomes in the Combined Public and GHRU Collection by Region and Country Income Class

Income Group and Region	No. of Countries Represented in Combined Public and GHRU Genome Collection (% of the Total Number of Countries in Each Income Group)	No. of Genomes Represented in Combined Public and GHRU Genome Collection (% of the Total Number of Genomes Across All Income Groups)
High income (80 countries)		
East Asia and Pacific	7	904
Europe and Central Asia	26	4452
Middle East and North Africa	6	795
North America	2	4970
Subtotal	41/80 (51.3%)	11 121 (69.1%)
Upper middle income (60 countries)		
East Asia and Pacific	3	1,213
Europe and Central Asia	8	449
Latin America and Caribbean	6 (incl. Colombia)	645 (incl. 493 GHRU)
Middle East and North Africa	2	3
Sub-Saharan Africa	1	36
Subtotal	20/60 (33.3%)	2346 (14.6%)
Lower middle income (47 countries)		
East Asia and Pacific	6 (incl. Philippines)	1186 (incl. 512 GHRU)
Latin America and Caribbean	1	3
Middle East and North Africa	1	37
South Asia	2 (incl. India)	577 (incl. 307 GHRU)
Sub-Saharan Africa	5 (incl. Nigeria)	374 (incl. 139 GHRU)
Subtotal	15/47 (31.9%)	2177 (13.5%)
Low income (31 countries)		
South Asia	2	117
Sub-Saharan Africa	4	323
Subtotal	6/31 (19.3%)	440 (2.7%)
Other	2	2
Total	84	16 086

Abbreviations: GHRU, Global Health Research Unit; incl., including.

### Species Identification

Pathogenwatch first assigns genome assemblies to a species via the Speciator tool. The assigned species then determines the downstream analyses. Speciator can currently identify assemblies belonging to *K. pneumoniae*, *K. quasipneumoniae*, *K. variicola*, *K. quasivariicola*, and *K. africana* (which together make up the *K. pneumoniae* species complex), as well as 11 other *Klebsiella* species (Supplementary Table 7).

*Klebsiella pneumoniae* accounted for 88.5% (14 635/16 537) and 88.7% (1451/1636) of *Klebsiella* genomes from the public and GHRU collections, respectively (Supplementary Table 8), reaffirming the clinical dominance of this species. The other most frequently observed species were *K. quasipneumoniae*, *K. variicola*, *K. aerogenes*, and *K. michiganensis* (comprising 4.0%, 1.9%, 1.8%, and 1.8% of the combined collections, respectively). As shown previously by others, we found inaccuracies in laboratory identification methods for *Klebsiella* [45]. For example, of the 1576 isolates in the GHRU collection assigned to *K. pneumoniae* using laboratory methods, Speciator identified 147 (9.3%) as *K. quasipneumoniae*, 4 (0.3%) as *K. variicola*, 2 (0.1%) as *K. michiganensis*, 2 (0.1%) as *K. oxytoca*, and 2 (0.1%) as *K. quasivariicola* (Supplementary Table 9).

### Surveillance of High-Risk Clones

Assemblies identified by Pathogenwatch as *Klebsiella* are subject to MLST and/or cgMLST based on the availability of schemes (Supplementary Table 7). The majority of our GHRU *K. pneumoniae* genomes belonged to a small number of known epidemic (“high risk”) sequence types (STs) that were also overrepresented in the public genome collection. In particular, 51.7% (750/1451) of the GHRU genomes and 56.7% (8295/14 635) public genomes belonged to only 10 STs that were the most frequently observed across the combined collections (Table 2). Overall, a high number of STs were observed in both collections (209 and 1115 in the GHRU and public collections, respectively). We also found 62 STs present among GHRU genomes that were not identified among public genomes, of which 33 were novel.

**Table 2.** Characteristics of the Top 10 Most Frequently Observed Sequence Types of *Klebsiella pneumoniae* in the Combined Public and GHRU Genome Collections

ST	No. (%) of Genomes in Public Collection	No. (%) of Genomes in GHRU Collection	Total No. (%) of Genomes in Both Collections	Total No. of Countries	Total (%) With a Carbapenemase	Most Frequently Observed Carbapenemases (≥10% Isolates)	Total (%) With an ESBL	Most Frequently Observed ESBLs (≥10% Isolates)	Median No. of Virulence Determinants per Genome (Range)	Most Frequently Observed Virulence Determinants (≥10% Isolates)^a^	Total No. of K-Types (and Types With ≥10% Isolates)^b^	Total No. of O-Types (and Types With ≥10% Isolates)^b^
11	1160 (7.9%)	87 (6.0%)	1247 (7.8%)	50	1049 (84.1%)	KPC-2 - 605 (48.5%); NDM-1 - 188 (15.1%); OXA-48 - 156 (12.5%)	1055 (84.6%)	CTX-M-15 - 523 (41.9%); CTX-M-65 - 360 (28.9%)	1 (0–5)	Yersiniabactin—1045 (83.8%)	28 (KL105, 16.2%; KL24, 16.4%; KL47, 14.8%; KL64, 27.9%)	8 (O2v1, 46.8%; O2v2, 20.2%; OL101, 13.2%)
14	293 (2.0%)	53 (3.7%)	346 (2.2%)	34	201 (58.1%)	NDM-1 - 107 (30.9%); OXA-232 - 68 (19.7%)	244 (70.5%)	CTX-M-15 - 232 (67.1%)	1 (0–4)	Yersiniabactin—235 (67.9%)	4 (KL2, 88.3%)	4 (O1v1, 94.1%)
15	802 (5.5%)	52 (3.6%)	854 (5.3%)	54	458 (53.6%)	NDM-1 - 169 (19.8%); OXA-48 - 112 (13.1%); KPC-2 - 101 (11.8%)	680 (79.6%)	CTX-M-15 - 627 (73.4%)	1 (0–4)	Yersiniabactin—390 (44.7%); aerobactin—105 (12.3%)	24 (KL112, 41.3%; KL24, 30.8%)	7 (O1v1, 88.0%)
16	433 (3.0%)	20 (1.4%)	453 (2.8%)	26	326 (72.0%)	OXA-232 - 192 (42.4%); NDM-1 - 158 (34.9%)	399 (88.1%)	CTX-M-15 - 390 (86.1%)	1 (0–3)	Yersiniabactin—278 (61.4%)	8 (KL51, 94.2%)	5 (O3b, 95.1%)
101	481 (3.3%)	22 (1.5%)	503 (3.1%)	43	353 (70.2%)	OXA-48 - 221 (43.9%); NDM-1 - 71 (14.1%)	414 (82.3%)	CTX-M-15 - 368 (73.2%)	1 (0–4)	Yersiniabactin—444 (88.3%)	3 (KL17, 95.4%)	3 (O1v1, 91.0%)
147	426 (2.9%)	197 (13.6%)	623 (3.9%)	42	474 (76.1%)	NDM-1 - 214 (34.3%); OXA-181 - 92(14.8%); OXA-48 - 79 (12.7%)	538 (86.4%)	CTX-M-15 - 506 (81.2%)	1 (0–4)	Yersiniabactin—378 (60.7%)	17 (KL10, 20.5%; KL64, 71.7%)	10 (O2v1, 74.1%; O3/O3a, 19.6%)
231	199 (1.4%)	108 (7.4%)	307 (1.9%)	19	255 (83.1%)	OXA-232 - 231 (75.2%)	226 (73.6%)	CTX-M-15 - 217 (70.7%)	2 (0–2)	Yersiniabactin—292 (95.1%); aerobactin—239 (77.9%)	2 (KL51, 97.8%)	4 (O1v2, 95.6%)
258	2936 (20.1%)	122 (8.4%)	3058 (19.0%)	31	2848 (93.1%)	KPC-2 - 1441 (47.1%); KPC-3 - 1391 (45.5%)	815 (26.7%)	SHV-12 - 630 (20.6%)	0 (0–4)	Yersiniabactin—1279 (41.8%); colibactin—870 (28.4%)	10 (KL106, 45.7%; KL107, 52.0%)	5 (O2v2, 98.4%)
307	984 (6.7%)	55 (3.8%)	1039 (6.5%)	45	442 (42.5%)	KPC-2 - 269 (25.9%)	973 (93.6%)	CTX-M-15 - 969 (93.3%)	0 (0–4)	Yersiniabactin—144 (13.9%)	1 (KL102, 100%)	3 (O2v2, 99.2%)
512	581 (4.0%)	34 (2.3%)	615 (3.8%)	22	612 (99.5%)	KPC-3 - 612 (99.5%)	10 (1.6%)	-	0 (0–2)	-	1 (KL107, 100%)	2 (O2v2, 99.5%)

Abbreviations: GHRU, Global Health Research Unit; ST, sequence type.

*All virulence determinants detected by Kleborate were included, regardless of completeness.

**Included K- and O-loci with confidence levels of “good” or above.

Clonal lineages of *K. pneumoniae* differ in their ability to acquire resistance and virulence genes, and in their propensity to spread within hospital and community environments [46]. Closing geographic gaps in genomic surveillance to fully describe the diversity of clones circulating across different regions is therefore vital for a better understanding of the local epidemiology of *K. pneumoniae* infections. We found clear differences in the dominant high-risk STs of *K. pneumoniae* circulating in the GHRU countries, with the single largest contribution from ST258 in Colombia (24.3%), ST231 in India (34.5%), ST307 in Nigeria (15.1%), and ST147 in the Philippines (20.1%) (Supplementary Figure 2). Each of these STs is widely disseminated across each country. Despite differences in sampling strategies between countries, these results are in line with previous observations relating to the regional distribution of high-risk STs [46–50].

**Figure 2.**
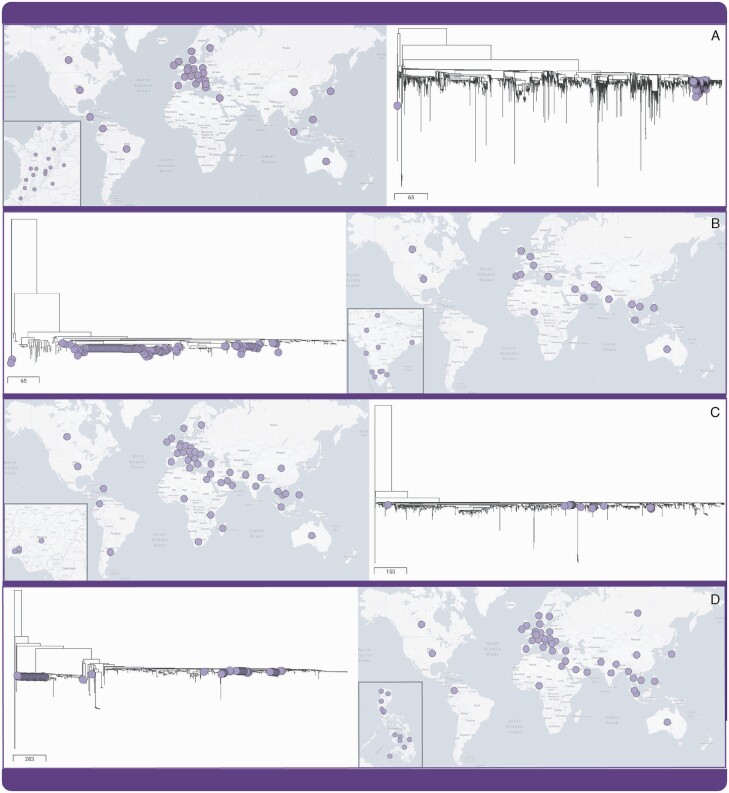
Pathogenwatch shows different dynamics of transmission and dissemination of the dominant “high risk” lineages in each GHRU participant country. ST258 genomes from Colombia form 1 main phylogenetic cluster, suggestive of a single successful introduction (*A*). ST231 genomes from India (*B*), ST307 genomes from Nigeria (*C*), and ST147 genomes from the Philippines (*D*) all form multiple phylogenetic clusters, suggesting multiple origins. The map insets show the widespread distribution of these clones in each country. Abbreviations: GHRU, Global Health Research Unit; ST, sequence type.

The ability of Pathogenwatch to generate phylogenetic trees of *K. pneumoniae* linked to metadata and other analytics provides a platform for monitoring the spread of lineages, offering relevant insights at global through to local scales. For example, ST258 genomes from Colombia form a single major cluster within the global phylogeny, which represents isolates from 31 countries (Figure 2A). This indicates that a single main introduction followed by within-country spread is likely responsible for the high endemicity of this lineage reported in Colombia [51]. By contrast, multiple phylogenetic clusters of ST231, ST307, and ST147 are observed in India, Nigeria, and the Philippines, respectively, demonstrating different origins for the multiple circulating lineages (Figure 2B–D). At a local scale, we found evidence of clonal spread of OXA-181– and CTX-M-15–producing ST147 within a hospital in India, over a period of 3 years (Supplementary Figure 3). However, some ST147 isolates collected from the same hospital during this period were phylogenetically distinct from the outbreak cluster, and thus the respective patients could be ruled out of the outbreak.

### Detection of Resistance and Virulence Mechanisms

Known resistance and virulence loci are identified in Pathogenwatch via Kleborate [28]. Resistance mechanisms currently include SNPs, acquired genes, and gene truncations that are relevant for different antibiotics or antibiotic classes. Virulence genes include those encoding acquired siderophores (yersiniabactin, salmochelin, aerobactin), the genotoxin colibactin, the hypermucoidy locus *rmpADC*, and alternative hypermucoidy marker gene *rmpA2*.

A high proportion of GHRU *K. pneumoniae* isolates contained an ESBL or carbapenemase gene (75.2% and 63.0%, respectively). This was also seen in the public genomes, with 54.5% (7983/14 635) of *K. pneumoniae* genomes carrying an ESBL and 57.5% (8416/14 635) carrying a carbapenemase. These rates far exceed those observed in most clinical settings to date (ie, typically <20% for carbapenemase-producing isolates) and demonstrate the tendency to prioritize multidrug-resistant isolates for sequencing [41, 52–54].

Despite sampling biases, clear differences existed between *K. pneumoniae* genomes from the GHRU countries with regard to major carbapenemase genes circulating (eg, KPC genes dominate in Colombia, NDM genes in the Philippines and Nigeria, OXA-48–like genes in India [Supplementary Figure 4]). These findings are in line with broader regional patterns reported previously and also uncovered using the public genomes [55–58]. In contrast, CTX-M-15 was consistently the most frequently observed ESBL gene, carried by 72.9–100% of ESBL-producing *K. pneumoniae* in each country.

Assessment of the prevalence and dissemination of mobile colistin resistance (*mcr*) genes, the first variant of which was discovered in 2015, among *K. pneumoniae* genomes in the public and GHRU collections revealed that these are still rare across all regions [59]. Only 0.3% (5/1636) of GHRU genomes carried an *mcr* gene, and 0.9% (128/14 635) of public genomes did. Among the latter, 52.3% (67/128) carried *mcr-9*.

**Figure 3.**
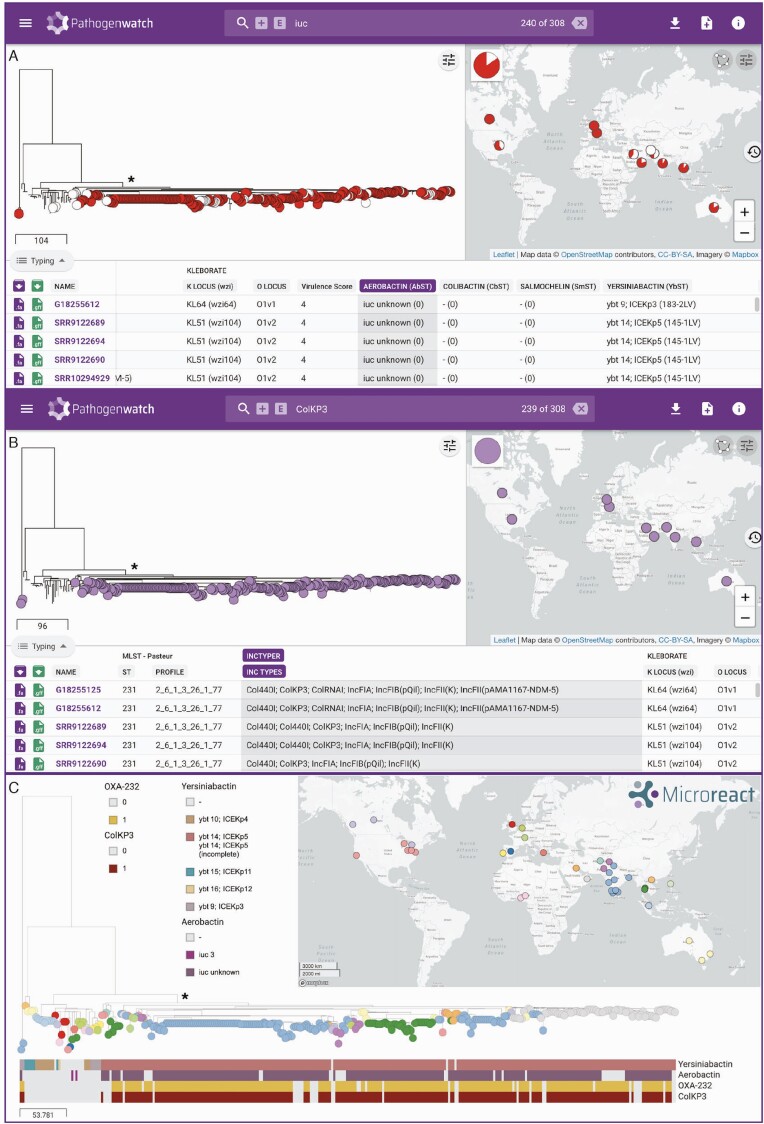
Pathogenwatch demonstrates convergence of virulence and resistance in a phylogenetic tree of 308 ST231 genomes from the public and GHRU collections (clade indicated with an asterisk (*). (*A*) The tree and map are filtered via the search bar by the presence of the virulence determinant aerobactin (*iuc*). All aerobactin-positive isolates are indicated with a circular node in the tree (red or white). Red nodes indicate the additional presence of the OXA-232 carbapenemase gene. Pie charts on the map show the relative proportion of aerobactin-positive isolates with and without OXA-232. (*B*) The tree and map are filtered via the search bar by the presence of replicon sequence ColKP3. ColKP3-positive isolates are indicated with purple nodes in the tree. (*C*) Likely acquisition of virulence loci (yersiniabactin and aerobactin) and plasmid-borne resistance (OXA-232 and ColKP3) followed by clonal expansion of the clade indicated with an asterisk (*). Abbreviations: GHRU, Global Health Research Unit; ST, sequence type.

We found that the majority (>80%) of all *K. pneumoniae* genomes in both public and GHRU genome collections had either no known acquired virulence factors or yersiniabactin only (Supplementary Table 10). However, there was an overrepresentation of colibactin among *K. pneumoniae* GHRU genomes from Colombia (present in 24.7% of isolates), which was associated with ST258. Furthermore, 37.5% of *K. pneumoniae* GHRU genomes from India carried aerobactin, which was almost always carried by ST231 or ST2096. In particular, phylogenetic analysis using a collection of ST231 isolates from both the GHRU and public collections highlighted a sublineage that has acquired aerobactin and yersiniabactin, as well as the OXA-232 carbapenemase (Figure 3A; see also [38]). This convergence of both resistance and virulence has been coupled with rapid clonal expansion and international spread, and close monitoring is needed.

### Monitoring of Mobile Genetic Elements

Plasmid replicons in any *Klebsiella* genome are identified in Pathogenwatch using the Inctyper tool. While it is not usually possible to directly link resistance or virulence genes to particular plasmids with short-read assemblies, we can nevertheless gain important epidemiological insights by analyzing patterns in the diversity and distribution of plasmid replicons.

For example, we noted that 68.7% (211/307) of GHRU *K. pneumoniae* isolates from India carry the ColKP3 replicon, which was not found in isolates from any of the other 3 countries. This replicon was previously found in a conserved 6.1-kb ColE-type plasmid initially reported from or linked to international travel to India but since identified elsewhere in patients without travel history and causing local outbreaks [60–63]. Of the 211 isolates with a ColKP3 replicon, all but one carry either the OXA-232 (*n*=168; 79.6%) or OXA-181 (*n*=42; 19.9%) carbapenemase.

Using the combined collection of GHRU and public *K. pneumoniae* genomes, we confirmed a strong association between the ColKP3 plasmid and the OXA-232 gene. In particular, we found that 86.7% (684/789) of *K. pneumoniae* isolates with a ColKP3 plasmid possess an OXA-232 gene, compared to 0.2% (35/15 297) of those without (Pearson’s chi-square=13 136.39; *P*<.0001). The association between ColKP3 and OXA-232 does not appear to be an artefact of lineage or geographic effects, as we found ColKP3/OXA-232 isolates in 45 different STs and 18 different countries overall. However, the majority (79.7%) do belong to only 4 STs (14, 16, 231, 2096), and they originate mostly from South and Southeast Asia, and the Arabian Peninsula. It has previously been suggested that disruption of the IS*Ecp1* transposase may have stabilized the OXA-232 gene on the ColKP3 plasmid [60]. Phylogenetic analysis of all ST231 isolates in Pathogenwatch suggested that the ColKP3 plasmid was acquired once, and then disseminated vertically through the lineage via clonal spread (Figure 3B and 3C; see also [38]).

### K- and O-Loci Monitoring to Aid Vaccine Development

O-antigen biosynthesis loci (O-loci) and capsular loci (including the *wzi* alleles and K-loci) present in *Klebsiella* genomes are identified in Pathogenwatch via Kleborate. Studies have reported development of a *K. pneumoniae* (and *Pseudomonas aeruginosa*) glycoconjugate vaccine based on the O-serotypes O1, O2, O3, and O5, and another for hypervirulent *K. pneumoniae* based on the K1 and K2 capsule types [19, 20]. It is thus crucial to monitor the diversity of O- and K-types across different lineages, geographic regions, age groups, clinical sources, and over time to ensure that a potential vaccine will adequately protect target populations.

Despite the biases present in both the public and GHRU sample collections, the breadth of geographic representation and inclusion of countries previously underrepresented make it a valuable collection to describe the diversity of O- and K-types. Here we considered human-associated isolates from the combined public and GHRU genomes that had O-types and K-types assigned with a confidence level of “good” or better by Kleborate.

We found that the O1, O2, and O3 serotypes (including their subtypes) were the most prevalent, comprising 88.9% (10 252/11 530) of *K. pneumoniae* isolates, and in line with previous reports [21]. Major high-risk STs with high levels of multidrug resistance were also dominated by these serotypes (Table 2). Other serotypes present in more than 1% of *K. pneumoniae* isolates included O4 (5.6%), OL101 (2.7%), and O5 (1.9%). As vaccines may be developed to target high-risk populations such as neonates, we also stratified the O-types identified in the GHRU isolates by patient age. We found that O1, O2, and O3 represented 52.9–91.4% of *K. pneumoniae* isolates from each age group (Supplementary Figure 5). Furthermore, we noted that the distribution of O-types varied substantially across species. For example, despite dominating in *K. pneumoniae* (88.9%), O1–O3 together made up only 40.5% (145/358) and 49.7% (94/189) of isolates from *K. quasipneumoniae* and *K. variicola*, respectively. Meanwhile, O5 was far more prevalent in both species (found in 29.9% and 40.7% isolates, respectively) than in *K. pneumoniae* (1.9%).

In contrast with the O-types, the 5 most common K-loci (KL) types (KL107, KL106, KL102, KL64, and KL51) represented only 47.8% (5218/10 922) of *K. pneumoniae* isolates, and a minimum of 39 KL-types were required to encompass 90% or more genomes. High-risk multidrug-resistant lineages were typically dominated by just 1 or 2 KL-types, although high numbers of types in some lineages also illustrate the capacity for genetic exchange of the *cps* locus. We found that the most frequently observed K-loci were not consistently present across all age groups sampled in the GHRU collection (Supplementary Figure 5), although larger sample collections of target populations with consistent sampling will be required for a robust assessment.

Likewise, future analyses using representative sample collections from target populations will further elucidate key trends and differences in K- and O-loci diversity. The importance of combining patient information with genomic data for developing insights relevant to patient outcomes cannot be overstated. The ability of Pathogenwatch to combine these data with the temporal and spatial trends of clonal lineages, and multidrug resistance and virulence, provides a rational system for informing the development of vaccines and therapeutics, and also for monitoring population changes as a consequence of implementing interventions.

### Concluding Remarks

Whole-genome sequencing empowers AMR surveillance laboratories to make public health decisions by providing a high-resolution view of the circulating bacterial strains and aiding outbreak investigations. Here we have presented the features of Pathogenwatch, a free, accessible platform for characterization and contextualization of *Klebsiella* genomes to aid surveillance at local, national, and global levels. The newly built capacity and expertise of 4 laboratories in LMICs to undertake ongoing genome sequencing, as developed during the wider GHRU project, will be enhanced by the use of Pathogenwatch and the increased representation of genomes from their countries. Extending this model to laboratories in other LMICs is a future priority.

## Supplementary Data

Supplementary materials are available at *Clinical Infectious Diseases* online. Consisting of data provided by the authors to benefit the reader, the posted materials are not copyedited and are the sole responsibility of the authors, so questions or comments should be addressed to the corresponding author.

ciab784_suppl_Supplementary_Material

ciab784_suppl_Supplementary_Table_2

ciab784_suppl_Supplementary_Table_3

ciab784_suppl_Supplementary_Table_5

ciab784_suppl_Supplementary_Table_6

## Notes

We are grateful to the DNA Pipelines and Pathogen Informatics teams at the Wellcome Sanger Institute for their support.

***Disclaimer.*** The views expressed in this publication are those of the authors and not necessarily those of the National Health Service, the National Institute for Health Research, or the Department of Health.

***Supplement sponsorship.*** The supplement is sponsored by the UK National Institute for Health Research Global Health Research Unit on Genomic Surveillance of AMR.

***Financial support.*** This work was supported by Official Development Assistance (ODA) funding from the National Institute for Health Research (grant number 16_136_111).

This research was commissioned by the National Institute for Health Research using ODA funding.

***Potential conflicts of interest.*** I. N. O. reports grants and nonfinancial support from International Vaccine Institute (SETA Award to the University of Ibadan), grants from UK Medical Research Council (MRC) and the Department for International Development (I. Okeke, University of Ibadan—Pathogenic lineages of enteric bacteria in Nigeria MR/L00464X/1), and grants from the UK National Institute for Health Research (NIHR; Global Health Research Unit on Genomic Surveillance of Antimicrobial Resistance Consortium), during the conduct of the study; reports being a member of the Bill & Melinda Gates Foundation Surveillance Advisors Group (from 2019–current). S. B. reports institutional support from Institut Pasteur, Paris, during the conduct of the study. E. J. F. reports provision of reference data, generated as part of the SpARK project funded by the JPI-AMR (3rd call)—UK funder MRC, during the conduct of the study. M. A. reports support from Wellcome Connecting Science, during the conduct of the study. M. K. reports support from Centre for Genomic Pathogen Surveillance (CGPS), during the conduct of the study, and funding, travel support, and receipt of equipment, materials, drugs, medical writing, gifts, or other services from the NIHR, outside the submitted work. K. L. R. reports travel support grant from NIHR, UK, outside the submitted work. H. H. reports funding from UK NIHR (Global Health Research Unit on Genomic Surveillance of Antimicrobial Resistance Consortium), during the conduct of the study. The other authors report no potential conflicts. All authors have submitted the ICMJE Form for Disclosure of Potential Conflicts of Interest. Conflicts that the editors consider relevant to the content of the manuscript have been disclosed.

***Acknowledgments.*** Members of the NIHR Global Health Research Unit on Genomic Surveillance of Antimicrobial Resistance: Johan Fabian Bernal, Alejandra Arevalo, Maria Fernanda Valencia, and Erik C. D. Osma Castro of the Colombian Integrated Program for Antimicrobial Resistance Surveillance—Coipars, CI Tibaitatá, Corporación Colombiana de Investigación Agropecuaria (AGROSAVIA), Tibaitatá–Mosquera, Cundinamarca, Colombia; Geetha Nagaraj, Varun Shamanna, Vandana Govindan, Akshata Prabhu, D. Sravani, M. R. Shincy, Steffimole Rose, and K. N. Ravishankar of the Central Research Laboratory, Kempegowda Institute of Medical Sciences, Bengaluru, India; Anderson O. Oaikhena, Ayorinde O. Afolayan, Jolaade J Ajiboye, and Erkison Ewomazino Odih of the Department of Pharmaceutical Microbiology, Faculty of Pharmacy, University of Ibadan, Oyo State, Nigeria; Marietta L. Lagrada, Polle Krystle V. Macaranas, Agnettah M. Olorosa, June M. Gayeta, Melissa Ana L. Masim, and Elmer M. Herrera of the Antimicrobial Resistance Surveillance Reference Laboratory, Research Institute for Tropical Medicine, Muntinlupa, the Philippines; Ali Molloy, alimolloy.com; John Stelling, The Brigham and Women’s Hospital; and Carolin Vegvari, Imperial College London, London, United Kingdom.

## References

[1] Podschun R, Ullmann U. Klebsiella spp. as nosocomial pathogens: epidemiology, taxonomy, typing methods, and pathogenicity factors. Clin Microbiol Rev 1998; 11:589-603. 9767057 10.1128/cmr.11.4.589 PMC88898

[2] Ko WC, Paterson DL, Sagnimeni AJ, et al. Community-acquired Klebsiella pneumoniae bacteremia: global differences in clinical patterns. Emerg Infect Dis 2002; 8:160-6. 11897067 10.3201/eid0802.010025 PMC2732457

[3] Bagley ST. Habitat association of Klebsiella species. Infect Control 1985; 6:52-8. 3882590 10.1017/s0195941700062603

[4] Rock C, Thom KA, Masnick M, Johnson JK, Harris AD, Morgan DJ. Frequency of Klebsiella pneumoniae carbapenemase (KPC)-producing and non-KPC-producing Klebsiella species contamination of healthcare workers and the environment. Infect Control Hosp Epidemiol 2014; 35:426-9. 24602950 10.1086/675598 PMC4030386

[5] Huynh BT, Passet V, Rakotondrasoa A, et al. Klebsiella pneumoniae carriage in low-income countries: antimicrobial resistance, genomic diversity and risk factors. Gut Microbes 2020; 11:1287-99. 32404021 10.1080/19490976.2020.1748257 PMC7527070

[6] Selden R, Lee S, Wang WL, Bennett JV, Eickhoff TC. Nosocomial klebsiella infections: intestinal colonization as a reservoir. Ann Intern Med 1971; 74:657-64. 5559431 10.7326/0003-4819-74-5-657

[7] Afroza S. Neonatal sepsis—a global problem: an overview. Mymensingh Med J 2006; 15:108-14. 16467776 10.3329/mmj.v15i1.2

[8] Meatherall BL, Gregson D, Ross T, Pitout JD, Laupland KB. Incidence, risk factors, and outcomes of Klebsiella pneumoniae bacteremia. Am J Med 2009; 122:866-73. 19699383 10.1016/j.amjmed.2009.03.034

[9] Pendleton JN, Gorman SP, Gilmore BF. Clinical relevance of the ESKAPE pathogens. Expert Rev Anti Infect Ther 2013; 11:297-308. 23458769 10.1586/eri.13.12

[10] Singh L, Cariappa MP, Kaur M. Klebsiella oxytoca: an emerging pathogen? Med J Armed Forces India 2016; 72:59-61. 10.1016/j.mjafi.2016.05.002 PMC5192185 28050072

[11] Rodríguez-Medina N, Barrios-Camacho H, Duran-Bedolla J, Garza-Ramos U. Klebsiella variicola: an emerging pathogen in humans. Emerg Microbes Infect 2019; 8:973-88. 31259664 10.1080/22221751.2019.1634981 PMC6609320

[12] Mathers AJ, Crook D, Vaughan A, et al. *K. quasipneumoniae* provides a window into carbapenemase gene transfer, plasmid rearrangements, and patient interactions with the hospital environment. Antimicrob Agents Chemother 2019; 63:e02513-18. 30910889 10.1128/AAC.02513-18 PMC6535554

[13] Cassini A, Högberg LD, Plachouras D, et al. Attributable deaths and disability-adjusted life-years caused by infections with antibiotic-resistant bacteria in the EU and the European Economic Area in 2015: a population-level modelling analysis. Lancet Infect Dis 2019; 19:56-66. 30409683 10.1016/S1473-3099(18)30605-4 PMC6300481

[14] Rawat D, Nair D. Extended-spectrum β-lactamases in gram negative bacteria. J Glob Infect Dis 2010; 2:263-74. 20927289 10.4103/0974-777X.68531 PMC2946684

[15] Queenan AM, Bush K. Carbapenemases: the versatile beta-lactamases. Clin Microbiol Rev 2007; 20:440-58. 17630334 10.1128/CMR.00001-07 PMC1932750

[16] Shon AS, Bajwa RP, Russo TA. Hypervirulent (hypermucoviscous) Klebsiella pneumoniae: a new and dangerous breed. Virulence 2013; 4:107-18. 23302790 10.4161/viru.22718 PMC3654609

[17] Gu D, Dong N, Zheng Z, et al. A fatal outbreak of ST11 carbapenem-resistant hypervirulent Klebsiella pneumoniae in a Chinese hospital: a molecular epidemiological study. Lancet Infect Dis 2018; 18:37-46. 28864030 10.1016/S1473-3099(17)30489-9

[18] Shen D, Ma G, Li C, et al. Emergence of a multidrug-resistant hypervirulent *Klebsiella pneumoniae* sequence type 23 strain with a rare blaCTX-M-24-harboring virulence plasmid. Antimicrob Agents Chemother 2019; 63:e02273-18. 30602516 10.1128/AAC.02273-18 PMC6395898

[19] Hegerle N, Choi M, Sinclair J, et al. Development of a broad spectrum glycoconjugate vaccine to prevent wound and disseminated infections with Klebsiella pneumoniae and Pseudomonas aeruginosa. PLoS One 2018; 13:e0203143. 30188914 10.1371/journal.pone.0203143 PMC6126813

[20] Feldman MF, Mayer Bridwell AE, Scott NE, et al. A promising bioconjugate vaccine against hypervirulent Klebsiella pneumoniae. Proc Natl Acad Sci USA 2019; 116:18655-63. 31455739 10.1073/pnas.1907833116 PMC6744904

[21] Follador R, Heinz E, Wyres KL, et al. The diversity of Klebsiella pneumoniae surface polysaccharides. Microb Genom 2016; 2:e000073. 28348868 10.1099/mgen.0.000073 PMC5320592

[22] David S, Reuter S, Harris SR, et al; EuSCAPE Working Group; ESGEM Study Group. Epidemic of carbapenem-resistant Klebsiella pneumoniae in Europe is driven by nosocomial spread. Nat Microbiol 2019; 4:1919-29. 31358985 10.1038/s41564-019-0492-8 PMC7244338

[23] Argimón S, Yeats CA, Goater RJ, et al. A global resource for genomic predictions of antimicrobial resistance and surveillance of Salmonella Typhi at Pathogenwatch. Nat Commun 2021; 12:2879. 34001879 10.1038/s41467-021-23091-2 PMC8128892

[24] Sánchez-Busó L, Yeats CA, Taylor B, et al. A community-driven resource for genomic epidemiology and antimicrobial resistance prediction of Neisseria gonorrhoeae at Pathogenwatch. Genome Med 2021; 13:61. 33875000 10.1186/s13073-021-00858-2 PMC8054416

[25] Centre for Genomic Pathogen Surveillance. Pathogenwatch assembly pipeline. Available at: https://gitlab.com/cgps/ghru/pipelines/assembly. Accessed 14 June 2021.

[26] Centre for Genomic Pathogen Surveillance. Pathogenwatch speciator. Available at: https://cgps.gitbook.io/pathogenwatch/technical-descriptions/species-assignment/speciator. Accessed 14 June 2021.

[27] Ondov BD, Treangen TJ, Melsted P, et al. Mash: fast genome and metagenome distance estimation using MinHash. Genome Biol 2016; 17:132. 27323842 10.1186/s13059-016-0997-x PMC4915045

[28] Lam MMC, Wick RR, Watts SC, Cerdeira LT, Wyres KL, Holt KE. A genomic surveillance framework and genotyping tool for *Klebsiella pneumoniae* and its related species complex. Nat Commun 2021;4188. 34234121 10.1038/s41467-021-24448-3 PMC8263825

[29] Jolley KA, Bray JE, Maiden MCJ. Open-access bacterial population genomics: BIGSdb software, the PubMLST.org website and their applications. Wellcome Open Res 2018; 3:124. 30345391 10.12688/wellcomeopenres.14826.1 PMC6192448

[30] Wyres KL, Wick RR, Gorrie C, et al. Identification of Klebsiella capsule synthesis loci from whole genome data. Microb Genom 2016; 2:e000102. 28348840 10.1099/mgen.0.000102 PMC5359410

[31] Wick RR, Heinz E, Holt KE, Wyres KL, Diekema DJ. User-friendly capsule and lipopolysaccharide serotype prediction for Klebsiella genomes. J Clin Microbiol 2018; 56:e00197-18. 29618504 10.1128/JCM.00197-18 PMC5971559

[32] Institute Pasteur. bigsDB *Klebsiella* schemes. Available at: https://bigsdb.pasteur.fr/klebsiella/; https://pubmlst.org/organisms/klebsiella-aerogenes/; https://pubmlst.org/organisms/klebsiella-oxytoca/. Accessed 14 June 2021.

[33] Centre for Genomic Pathogen Surveillance. Pathogenwatch inctyper. Available at: https://cgps.gitbook.io/pathogenwatch/technical-descriptions/inctyper. Accessed 14 June 2021.

[34] Carattoli A, Zankari E, García-Fernández A, et al. In silico detection and typing of plasmids using PlasmidFinder and plasmid multilocus sequence typing. Antimicrob Agents Chemother 2014; 58:3895-903. 24777092 10.1128/AAC.02412-14 PMC4068535

[35] Page AJ, Cummins CA, Hunt M, et al. Roary: rapid large-scale prokaryote pan genome analysis. Bioinformatics 2015; 31:3691-3. 26198102 10.1093/bioinformatics/btv421 PMC4817141

[36] Camacho C, Coulouris G, Avagyan V, et al. BLAST plus: architecture and applications. BMC Bioinformatics. 2009; 10:421. 20003500 10.1186/1471-2105-10-421 PMC2803857

[37] Saavedra SY, Bernal JF, Montilla-Escudero E, et al. Complexity of genomic epidemiology of carbapenem-resistant klebsiella pneumoniae isolates in Colombia urges the reinforcement of whole genome sequencing-based surveillance programs. Clin Infect Dis 2021;73: In this supplement. 10.1093/cid/ciab777 PMC8634422 34850835

[38] Nagaraj G, Shammana V, Govindan V, et al. High-resolution genomic profiling of carbapenem-resistant *Klebsiella pneumoniae* isolates: a multicentric retrospective Indian study. Clin Infect Dis 2021;73: In this supplement. 10.1093/cid/ciab767 PMC8634558 34850832

[39] Afolayan AO, Oaikhena AO, Aboderin AO, et al. Clones and clusters of antimicrobial-resistant *Klebsiella* from southwestern Nigeria. Clin Infect Dis 2021;73: In this supplement. 10.1093/cid/ciab769 PMC8634535 34850837

[40] Carlos CC, Masim MAL, Lagrada ML, et al. Genome sequencing identifies previously unrecognized *Klebsiella pneumoniae* outbreaks in neonatal intensive care units in the Philippines. Clin Infect Dis 2021;73: In this supplement. 10.1093/cid/ciab776 PMC8634409 34850834

[41] Argimón S, Masim MAL, Gayeta JM, et al. Integrating whole-genome sequencing within the national antimicrobial resistance surveillance program in the Philippines. Nat Commun 2020; 11:2719. 32483195 10.1038/s41467-020-16322-5 PMC7264328

[42] Centre for Genomic Pathogen Surveillance. Pathogenwatch technical descriptions. Available at: https://cgps.gitbook.io/pathogenwatch/technical-descriptions/. Accessed 14 June 2021.

[43] The NIHR Global Health Research Unit (GHRU) on Genomic Surveillance of Antimicrobial Resistance and The Center for Genomic Pathogen Surveillance (CGPS). Pathogenwatch collection of 1451 *K. pneumoniae* genomes from GHRU participating countries. Available at: https://pathogen.watch/collection/f9tijvy5mn5f-ghru-k-pneumoniae. Accessed 14 June 2021.

[44] Temkin E, Fallach N, Almagor J, et al. Estimating the number of infections caused by antibiotic-resistant *Escherichia coli* and *Klebsiella pneumoniae* in 2014: a modelling study. Lancet Glob Health 2018; 6:e969-e79. 30103998 10.1016/S2214-109X(18)30278-X

[45] Long SW, Linson SE, Ojeda Saavedra M, et al. Whole-genome sequencing of human clinical *Klebsiella pneumoniae* isolates reveals misidentification and misunderstandings of *Klebsiella pneumoniae*, *Klebsiella variicola*, and *Klebsiella quasipneumoniae*. mSphere 2017; 2:e00290-17. 28776045 10.1128/mSphereDirect.00290-17 PMC5541162

[46] Wyres KL, Lam MMC, Holt KE. Population genomics of Klebsiella pneumoniae. Nat Rev Microbiol 2020; 18:344-59. 32055025 10.1038/s41579-019-0315-1

[47] Wyres KL, Nguyen TNT, Lam MMC, et al. Genomic surveillance for hypervirulence and multi-drug resistance in invasive Klebsiella pneumoniae from South and Southeast Asia. Genome Med 2020; 12:11. 31948471 10.1186/s13073-019-0706-y PMC6966826

[48] Peirano G, Chen L, Kreiswirth BN, Pitout JDD. Emerging antimicrobial-resistant high-risk *Klebsiella pneumoniae* clones ST307 and ST147. Antimicrob Agents Chemother 2020; 64:e01148-20. 32747358 10.1128/AAC.01148-20 PMC7508593

[49] Shankar C, Venkatesan M, Rajan R, et al. Molecular characterization of colistin-resistant Klebsiella pneumoniae & its clonal relationship among Indian isolates. Indian J Med Res 2019; 149:199-207. 31219084 10.4103/ijmr.IJMR_2087_17 PMC6563726

[50] Castanheira M, Costello AJ, Deshpande LM, Jones RN. Expansion of clonal complex 258 KPC-2-producing Klebsiella pneumoniae in Latin American hospitals: report of the SENTRY antimicrobial surveillance program. Antimicrob Agents Chemother 2012; 56:1668-9; author reply 1670-1. 22337899 10.1128/AAC.05942-11 PMC3294890

[51] Rojas LJ, Weinstock GM, De La Cadena E, et al. An analysis of the epidemic of Klebsiella pneumoniae carbapenemase-producing K. pneumoniae: convergence of two evolutionary mechanisms creates the “perfect storm”. J Infect Dis 2017; 217:82-92. 29029188 10.1093/infdis/jix524 PMC5853647

[52] Han JH, Goldstein EJ, Wise J, Bilker WB, Tolomeo P, Lautenbach E. Epidemiology of carbapenem-resistant klebsiella pneumoniae in a network of long-term acute care hospitals. Clin Infect Dis 2017; 64:839-44. 28013258 10.1093/cid/ciw856 PMC5399931

[53] Jesumirhewe C, Springer B, Lepuschitz S, Allerberger F, Ruppitsch W. Carbapenemase-producing *Enterobacteriaceae* isolates from Edo State, Nigeria. Antimicrob Agents Chemother 2017; 61:e00255-17. 28607027 10.1128/AAC.00255-17 PMC5527642

[54] Castagnola E, Tatarelli P, Mesini A, et al. Epidemiology of carbapenemase-producing enterobacteriaceae in a pediatric hospital in a country with high endemicity. J Infect Public Health 2019; 12:270-4. 30477920 10.1016/j.jiph.2018.11.003

[55] Pitout JDD, Peirano G, Kock MM, Strydom KA, Matsumura Y. The global ascendency of OXA-48-type carbapenemases. Clin Microbiol Rev 2019; 33:e00102-19. 31722889 10.1128/CMR.00102-19 PMC6860007

[56] Logan LK, Weinstein RA. The epidemiology of carbapenem-resistant Enterobacteriaceae: the impact and evolution of a global menace. J Infect Dis 2017; 215:28-36. 10.1093/infdis/jiw282 PMC5853342 28375512

[57] Nordmann P, Poirel L. The difficult-to-control spread of carbapenemase producers among Enterobacteriaceae worldwide. Clin Microbiol Infect 2014; 20:821-30. 24930781 10.1111/1469-0691.12719

[58] Munoz-Price LS, Poirel L, Bonomo RA, et al. Clinical epidemiology of the global expansion of Klebsiella pneumoniae carbapenemases. Lancet Infect Dis 2013; 13:785-96. 23969216 10.1016/S1473-3099(13)70190-7 PMC4673667

[59] Liu YY, Wang Y, Walsh TR, et al. Emergence of plasmid-mediated colistin resistance mechanism MCR-1 in animals and human beings in China: a microbiological and molecular biological study. Lancet Infect Dis 2016; 16:161-8. 26603172 10.1016/S1473-3099(15)00424-7

[60] Potron A, Rondinaud E, Poirel L, et al. Genetic and biochemical characterisation of OXA-232, a carbapenem-hydrolysing class D β-lactamase from Enterobacteriaceae. Int J Antimicrob Agents 2013; 41:325-9. 23305656 10.1016/j.ijantimicag.2012.11.007

[61] Shu L, Dong N, Lu J, et al. Emergence of OXA-232 carbapenemase-producing *Klebsiella pneumoniae* that carries a pLVPK-like virulence plasmid among elderly patients in China. Antimicrob Agents Chemother 2019; 63:e02246-18. 30559135 10.1128/AAC.02246-18 PMC6395905

[62] Espinal P, Nucleo E, Caltagirone M, et al. Genomics of *Klebsiella pneumoniae* ST16 producing NDM-1, CTX-M-15, and OXA-232. Clin Microbiol Infect 2019; 25:385.e1-5. 30472424 10.1016/j.cmi.2018.11.004

[63] Li X, Ma W, Qin Q, et al. Nosocomial spread of OXA-232-producing Klebsiella pneumoniae ST15 in a teaching hospital, Shanghai, China. BMC Microbiol 2019; 19:235. 31660869 10.1186/s12866-019-1609-1 PMC6819465

